# Supplementary material for: Ethical acceptability of human challenge trials: Consultation with the US public and with research personnel
Source: PLoS One. 2024 Oct 22;19(10):e0307808. doi: 10.1371/journal.pone.0307808 (PMC11495607; doi:10.1371/journal.pone.0307808)
Supplement: S1 File — File containing all supplementary figures and additional information on the analytic approach and raw questionnaire. (DOCX) [file pone.0307808.s001.docx]

**Supplementary materials for:**

**Ethical Acceptability of Human Challenge Trials: Consultation with the US public and with research personnel**

**Priors used in analyses**

For probability of superiority analyses, the following priors were used:

normal(0, 1), class = Intercept

normal(0, 1), class = b

exponential(2), class = sd

For analyses of categorical outcomes (e.g., Yes vs. No vs. No and I do not support any type of research involving humans), the following priors were used:

normal(0, 1), class = Intercept

normal(0, 1), class = b

exponential(2), class = sd

For analyses of Likert scale items, the following priors were used:

normal(0, 4), class = Intercept

normal(0, 1), class = b

exponential(4), class = sd

As noted in the manuscript, the pre-registered prior for Likert items was normal(0, 2) for intercepts but the prior was broadened, which we believe enabled the MCMC sampling to function better for some items where very few people gave certain ratings.

**Sensitivity to inclusion/exclusion according to non-binary sex identification**

In the main text, we noted that 25 respondents were excluded from analyses owing to identifying as nether male nor female: The official US data that we poststratify by does not include non-binary options, only allowing male vs. female. As such, we cannot poststratify towards any particular percentage of non-binary respondents in the general US population. It may be a concern that excluding these respondents would significantly bias the results, but we show below that this seems to not be the case. In a set of sensitivity analyses we assigned these respondents to either being all male or all female and then included them in analyses for ratings of general support, importance, and acceptability. In all cases, there was no impact of these respondents being included or excluded.


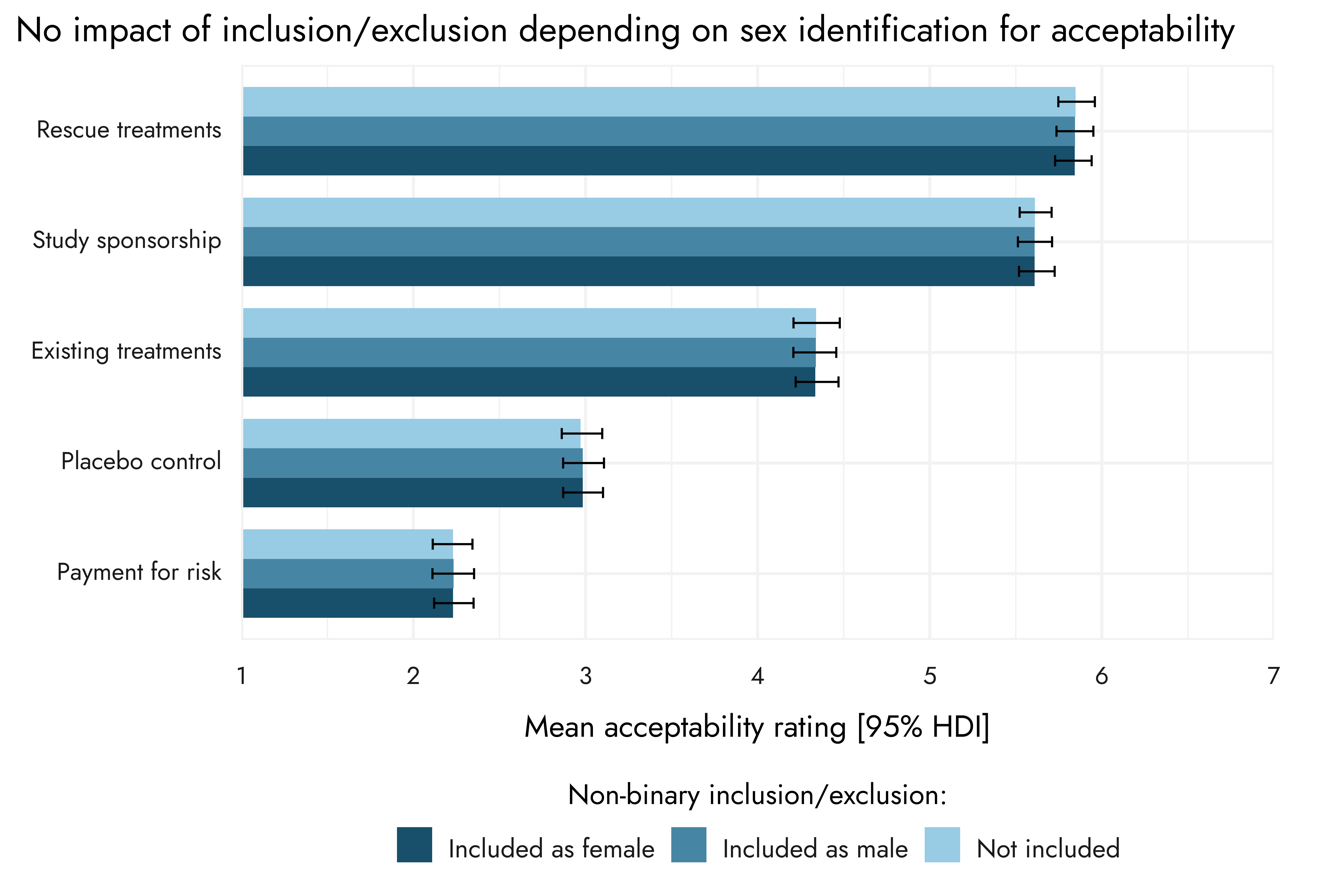

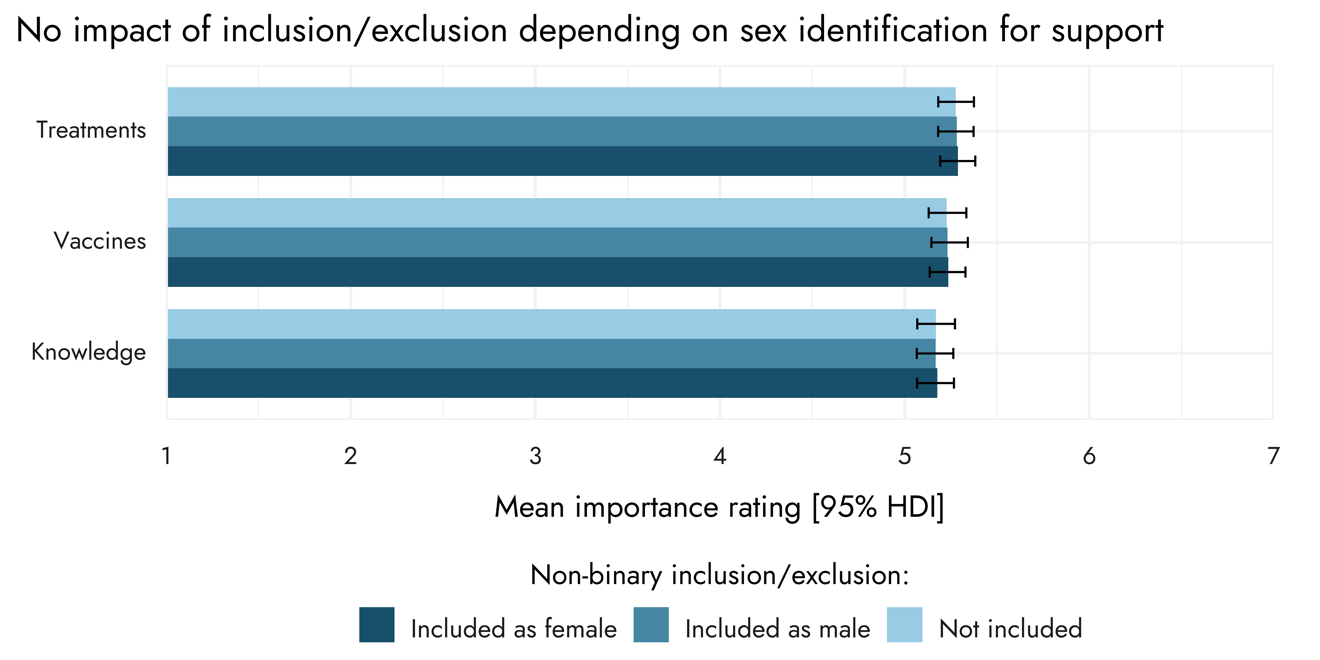
Figure S1. Non-binary identification inclusion/exclusion has no impact on support.

Figure S2. Non-binary identification inclusion/exclusion has no impact on importance
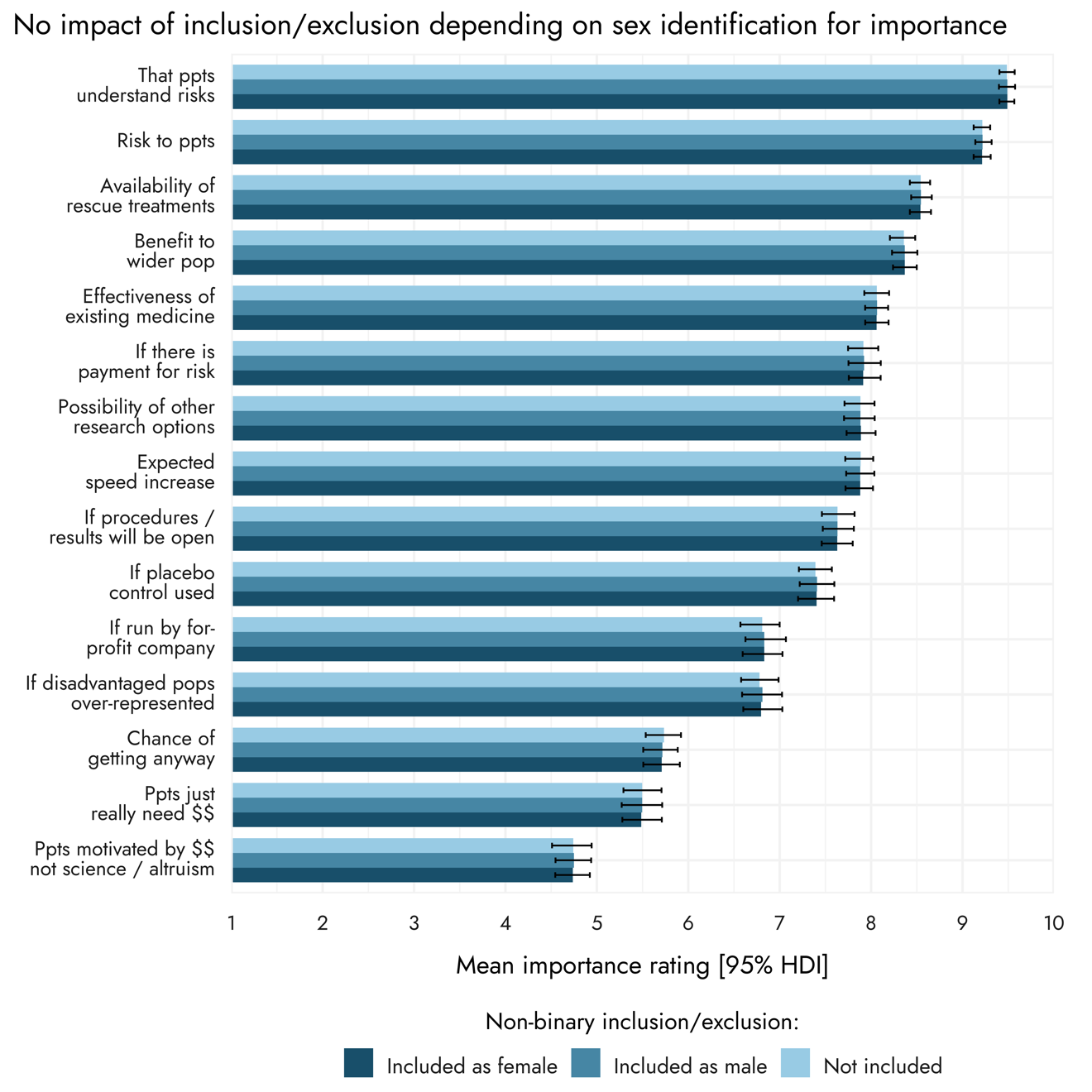


**Sample information for research personnel across different outcomes**

The total number of eligible research personnel was 33.

Table S1. Sample information for research personnel

|  | Importance | | Allow HCTs | | Acceptability | | Barriers | | Support | |
| --- | --- | --- | --- | --- | --- | --- | --- | --- | --- | --- |
| ***PhD in field*** | *n* | *%* | *n* | *%* | *n* | *%* | *n* | *%* | *n* | *%* |
| Yes | 22 | 66.7 | 14 | 70 | 22 | 68.8 | 21 | 70 | 15 | 71.4 |
| No | 10 | 30.3 | 6 | 30 | 10 | 31.2 | 9 | 30 | 6 | 28.6 |
| Missing | 1 | 3 | 0 | 0 | 0 | 0 | 0 | 0 | 0 | 0 |
|  |  |  |  |  |  |  |  |  |  |  |
| ***Main research country*** | *n* | *%* | *n* | *%* | *n* | *%* | *n* | *%* | *n* | *%* |
| USA | 8 | 24.2 | 8 | 40 | 8 | 25 | 8 | 26.7 | 8 | 38.1 |
| UK | 5 | 15.2 | 2 | 10 | 5 | 15.6 | 5 | 16.7 | 2 | 9.5 |
| Canada | 4 | 12.1 | 2 | 10 | 4 | 12.5 | 4 | 13.3 | 2 | 9.5 |
| Netherlands | 3 | 9.1 | 1 | 5 | 3 | 9.4 | 2 | 6.7 | 1 | 4.8 |
| Australia | 2 | 6.1 | 1 | 5 | 2 | 6.2 | 2 | 6.7 | 1 | 4.8 |
| Thailand | 2 | 6.1 | 1 | 5 | 2 | 6.2 | 2 | 6.7 | 1 | 4.8 |
| Vietnam | 2 | 6.1 | 2 | 10 | 2 | 6.2 | 2 | 6.7 | 2 | 9.5 |
| Missing | 2 | 6.1 | 1 | 5 | 1 | 3.1 | 1 | 3.3 | 1 | 4.8 |
| Chile | 1 | 3 | 1 | 5 | 1 | 3.1 | 1 | 3.3 | 1 | 4.8 |
| Italy | 1 | 3 | 0 | 0 | 1 | 3.1 | 1 | 3.3 | 1 | 4.8 |
| Malawi | 1 | 3 | 0 | 0 | 1 | 3.1 | 0 | 0 | 0 | 0 |
| Peru | 1 | 3 | 1 | 5 | 1 | 3.1 | 1 | 3.3 | 1 | 4.8 |
| Singapore | 1 | 3 | 0 | 0 | 1 | 3.1 | 1 | 3.3 | 0 | 0 |
|  |  |  |  |  |  |  |  |  |  |  |
| ***Type of research*** | *n* | *%* | *n* | *%* | *n* | *%* | *n* | *%* | *n* | *%* |
| Vaccines | 21 | 63.6 | 11 | 55 | 21 | 65.6 | 20 | 66.7 | 11 | 52.4 |
| Treatments | 17 | 51.5 | 11 | 55 | 17 | 53.1 | 17 | 56.7 | 12 | 57.1 |
| None of the above | 1 | 3 | 1 | 5 | 1 | 3.1 | 0 | 0 | 1 | 4.8 |
|  |  |  |  |  |  |  |  |  |  |  |
| ***Research context*** | *n* | *%* | *n* | *%* | *n* | *%* | *n* | *%* | *n* | *%* |
| Academic / Research hospital | 29 | 87.9 | 17 | 85 | 29 | 90.6 | 27 | 90 | 18 | 85.7 |
| Pharma | 4 | 12.1 | 3 | 15 | 4 | 12.5 | 4 | 13.3 | 3 | 14.3 |
|  |  |  |  |  |  |  |  |  |  |  |
| ***Research role*** | *n* | *%* | *n* | *%* | *n* | *%* | *n* | *%* | *n* | *%* |
| PI | 17 | 51.5 | 10 | 50 | 17 | 53.1 | 17 | 56.7 | 10 | 47.6 |
| Study coordinator | 5 | 15.2 | 3 | 15 | 5 | 15.6 | 4 | 13.3 | 4 | 19 |
| Admin or logistics | 4 | 12.1 | 2 | 10 | 4 | 12.5 | 3 | 10 | 2 | 9.5 |
| Statistician | 4 | 12.1 | 4 | 20 | 4 | 12.5 | 3 | 10 | 4 | 19 |
| Physician | 3 | 9.1 | 1 | 5 | 3 | 9.4 | 3 | 10 | 1 | 4.8 |
| Clinical trials manager | 2 | 6.1 | 2 | 10 | 2 | 6.2 | 2 | 6.7 | 2 | 9.5 |
| Data manager | 2 | 6.1 | 2 | 10 | 2 | 6.2 | 2 | 6.7 | 2 | 9.5 |
| Qualitative researcher | 2 | 6.1 | 0 | 0 | 2 | 6.2 | 1 | 3.3 | 0 | 0 |
| Recruitment coordinator | 2 | 6.1 | 1 | 5 | 2 | 6.2 | 2 | 6.7 | 1 | 4.8 |
| Device / pharmaceutical provision | 1 | 3 | 1 | 5 | 1 | 3.1 | 1 | 3.3 | 1 | 4.8 |

**Search information for expert sample**

We used PubMed to search for published articles and ClinicalTrials.gov to search for ongoing an completed Phase III trials between 2017 and 2023 for the following pathogens/diseases/conditions/interventions: bardotella pertussis, BCG, campylobacter, COVID-19/SARS CoV2, dengue, e-coli, hepatitis, HIV, hookworm, influenza, leishmaniasis, monkeypox, norovirus, pharyngitis, rabies, respiratory syncytial virus (RSV), rhinovirus, rotavirus, rubella, salmonella, schistosomiasis/schistosoma mansoni, shigella, streptococcus pneumoniae, tuberculosis, typhoid, cholera/vibrio cholerae, zika virus. For PubMed, search results were filtered to show only RCTs or clinical trials.

**Simulation-based assessments of false positive/false discovery rates**

**Bayesian *PSup* analyses in public opinion sample.** To assess the false positive rates from our Bayesian *PSup* comparisons, we need to perform multiple simulations of what might be observed if there were no differences between all the items. However, running full MRP for so many comparisons as are in the *Importance* ratings (105 when pairing all 15 items), and then for 100+ simulations was not practically feasible. Instead, we aimed to work with posterior estimates that could be seen as similar in width/level of uncertainty to the posterior estimates from MRP, but run much more rapidly. When centered around .5, the MRP Highest Density Intervals (HDIs) for *PSup* had widths of between .06 and .07. We found that generating *PSup* estimates from categorical regressions with sample sizes of 800 could produce posteriors with similar widths of HDI for a *PSup* around .5.
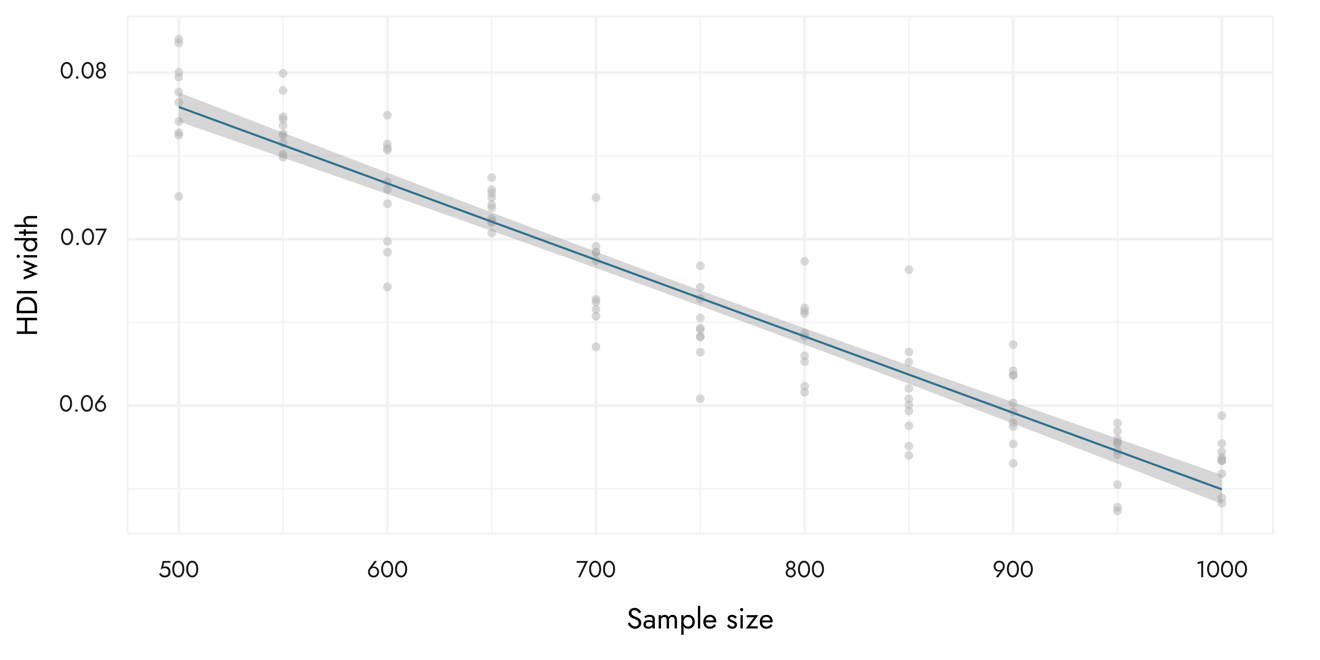


Figure S3. Probability of superiority highest density interval width depending on sample size.

We then performed 200 simulations of *Importance* ratings, with mean differences between the 15 *Importance* items at 0, and within subjects correlations between items at .25. Cutpoints with some random variation across simulations were used to convert the simulated, normally distributed data to 10-point Likert-style ratings, and paired comparisons were converted to a categorical format for regression as was done in the MRP analyses. In each simulation, posterior *PSup* estimates were then generated from the regression models for all 105 pairs of items. We found that with a simple point comparison of the HDI against a null of *PSup* = .5 would produce, on average across simulations, an error rate of 5.6%, whereas with a ROPE of .47-.53, false positives were largely eliminated, at 0.3%.


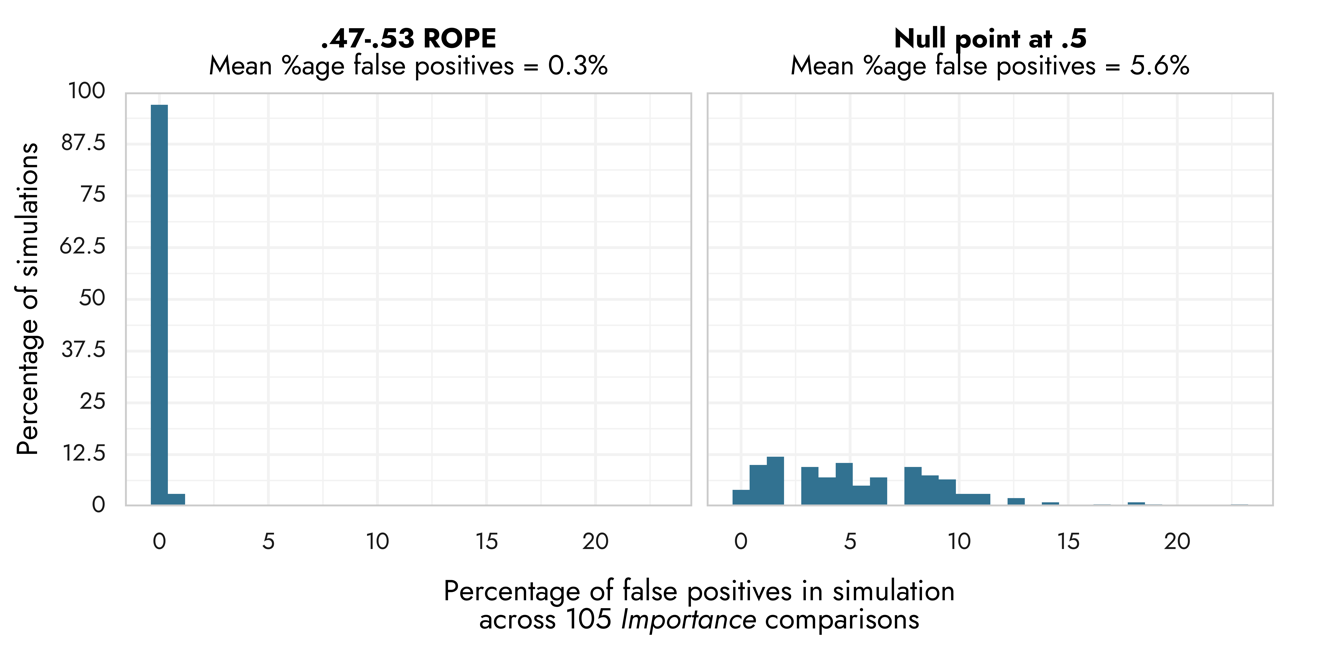
Figure S4. False positive rate depending on decision criteria for probability of superiority.

**Permutation testing of *PSup* in research personnel sample.** For research personnel, we simulated false positive rates for the permutation testing approach for comparisons among *Support* ratings, *Importance* ratings, and *HCT Barriers*. The approach to generate simulated data was the same as the Bayesian simulations above, again generating data that would on average have no differences among the item ratings. For *Support* ratings, we used a within subjects correlation of .75 to match the much higher expected (and observed) correlations among the different types of support, whereas we used a correlation of .25 for the other outcomes (*Support* ratings for different forms of HCT are understandably highly correlated, whereas correlations can vary a lot and tend to be much lower among the *Importance* ratings and *HCT barriers*). We also included an additional variation of how Likert ratings were thresholded, in case this yielded substantially different outcomes (by default we used thresholds that would tend to have a peak of responses around the middle of the Likert range, whereas in this case we added a version of threshold that were evenly distributed across Likert response options, with some random variation).


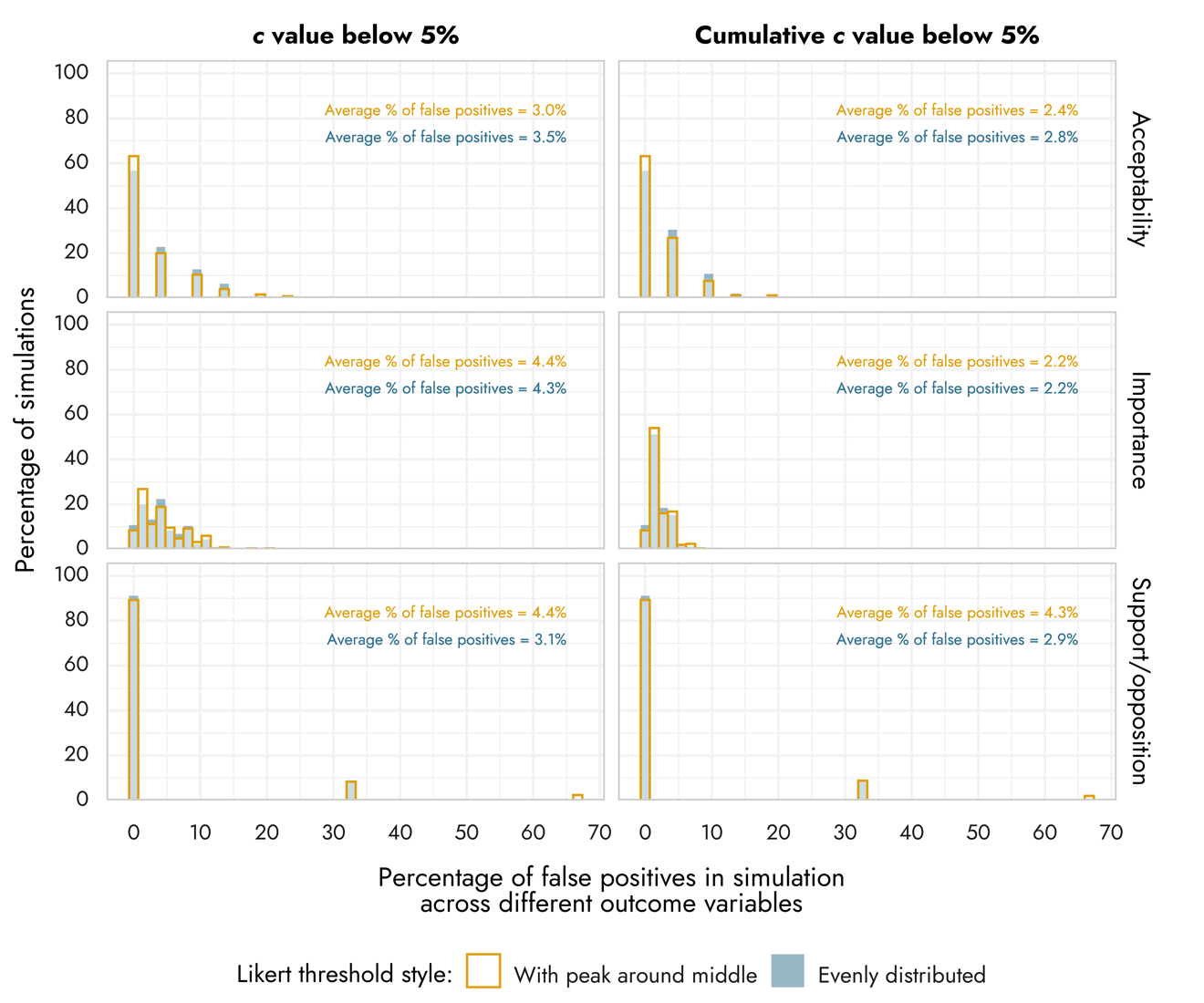


Figure S5. False positive rates for different decision criteria in research personnel.

We found that the rate of false positives across simulations did not vary very much depending on the type of thresholding used to determine the Likert scaling, and all approaches tended to keep the false discovery rate across simulations on average below 5%. Restricting selection of significant comparisons to only those that could be included in a cumulative chance percentage of 5% (ordering the chance values from smallest to largest and adding the chance values to one another until reaching a sum of 5%) tended to further reduce the average false positive rate, and was the option selected to summarize findings in the manuscript.

**Sample information for general population**

Table S2. Sample information for general population

|  | **Response** | **%age** | **n** |
| --- | --- | --- | --- |
| **Sex** | Female | 50% | 750 |
|  | Male | 50% | 750 |
|  |  |  |  |
| **Household income** | <$20k | 8.30% | 125 |
|  | $20-$49K | 28.50% | 427 |
|  | $50-$79K | 24.10% | 362 |
|  | $80-$99K | 12.60% | 189 |
|  | $100-$150K | 14.10% | 212 |
|  | >$150k | 8.30% | 125 |
|  |  |  |  |
| **Region** | Midwest | 20.80% | 312 |
|  | Northeast | 18.30% | 274 |
|  | South | 41% | 615 |
|  | West | 19.90% | 299 |
|  |  |  |  |
| **Education** | High school or less | 12.90% | 194 |
|  | Some college, no degree | 33.00% | 495 |
|  | Graduated from college | 38.50% | 578 |
|  | Completed grad school | 15.50% | 233 |
|  |  |  |  |
| **Party** | Democrat | 51.50% | 772 |
|  | Independent | 33.10% | 497 |
|  | Republican | 15% | 231 |
|  |  |  |  |
| **Race** | Asian or Asian American | 7.00% | 105 |
|  | Black or African American | 7.50% | 113 |
|  | Hispanic or Latino | 8.90% | 134 |
|  | Other | 3.00% | 45 |
|  | White or Caucasian | 73.50% | 1103 |
|  |  |  |  |
| **Age** | 18-24 | 9.70% | 146 |
|  | 25-34 | 29.50% | 442 |
|  | 35-44 | 26.50% | 397 |
|  | 45-64 | 27.70% | 416 |
|  | 65+ | 6.60% | 99 |

**Full plot of importance ratings**

**
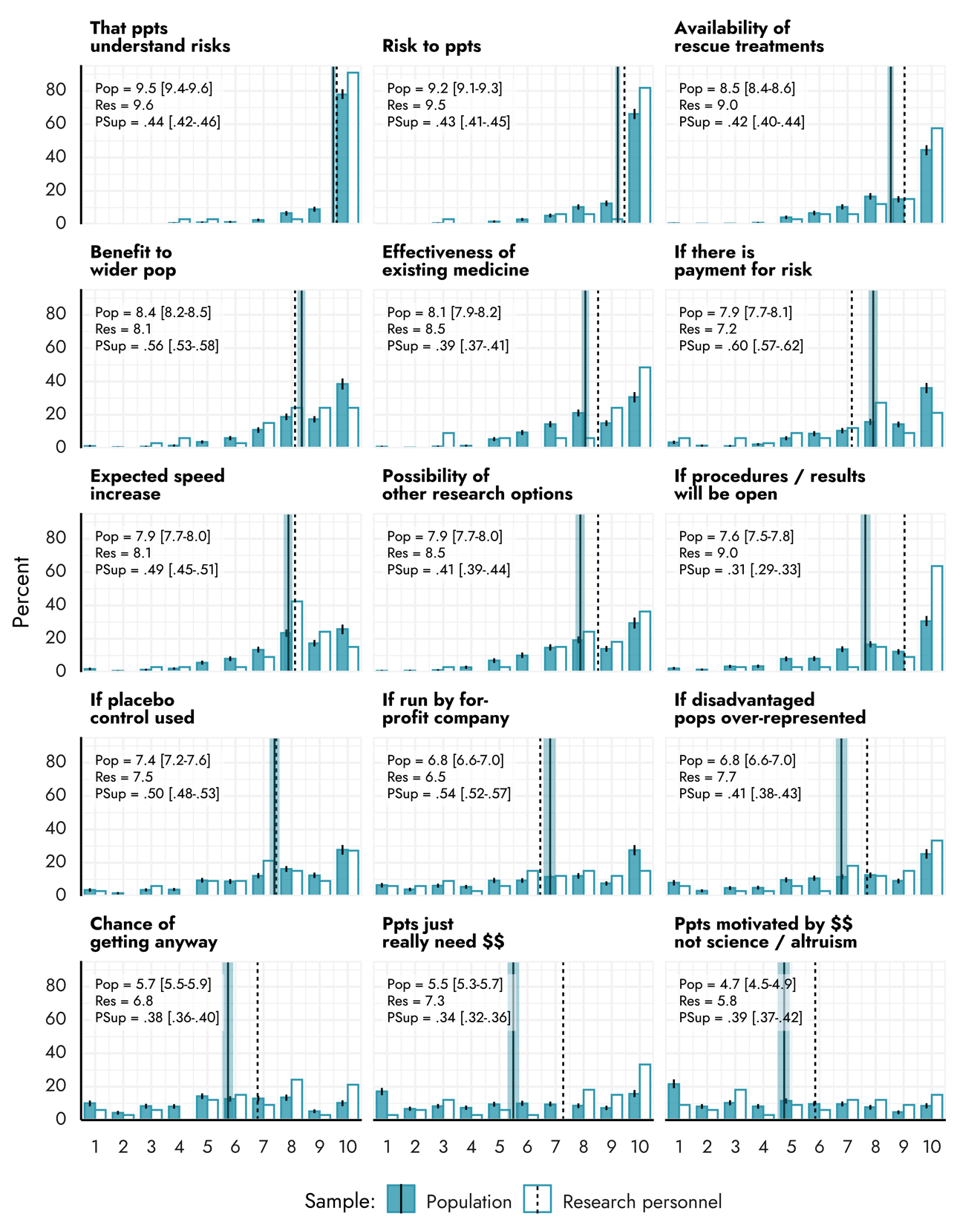
**

Figure S6. Full estimated responses for importance ratings.

**Full plot of *Placebo control* and *Trial sponsorship* acceptability**

**
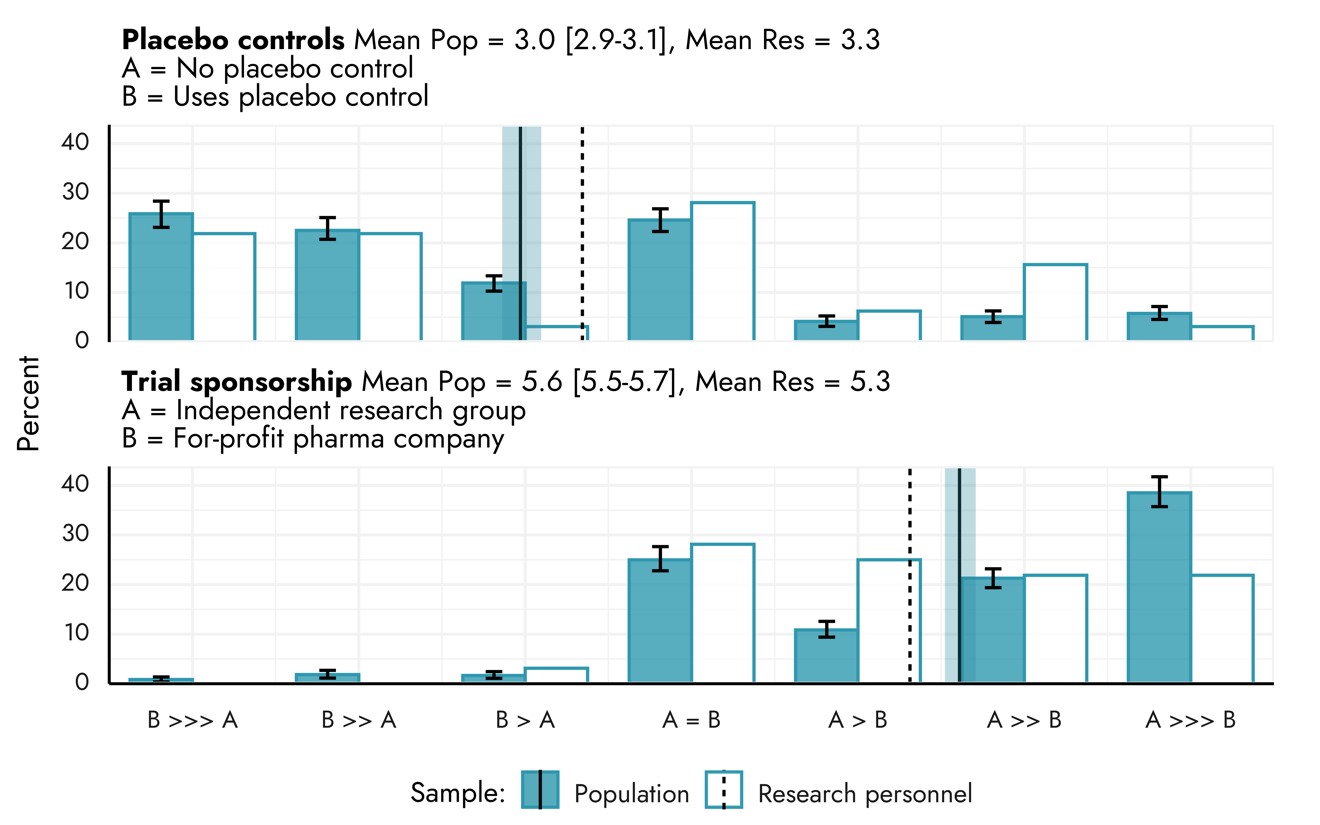
**

Figure S7. Full estimated responses for acceptability ratings.

**Full plot of research personnel barriers to conducting an HCT**

**
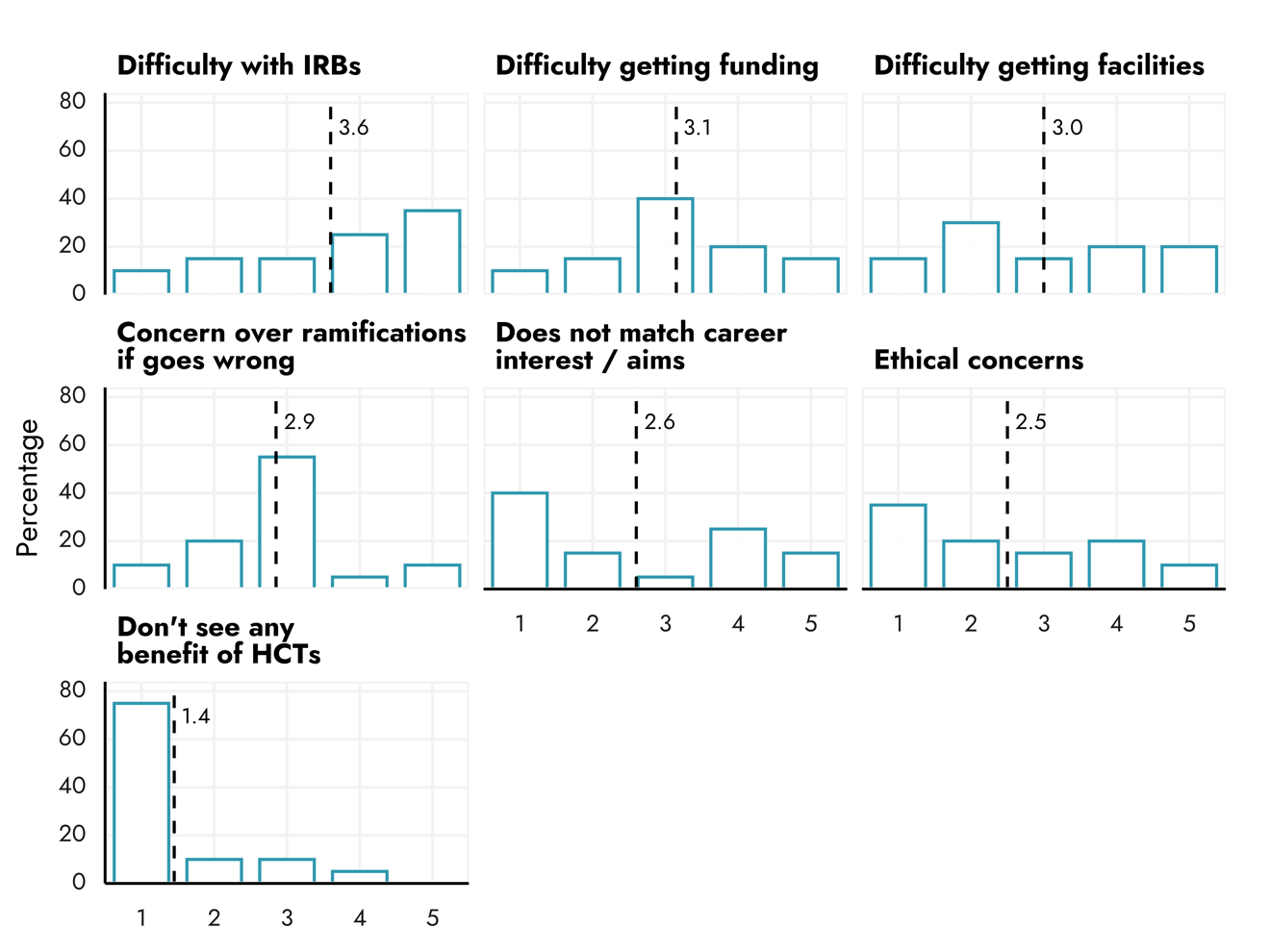
**

Figure S8. Full research personnel responses for barriers to conducting an HCT.

**
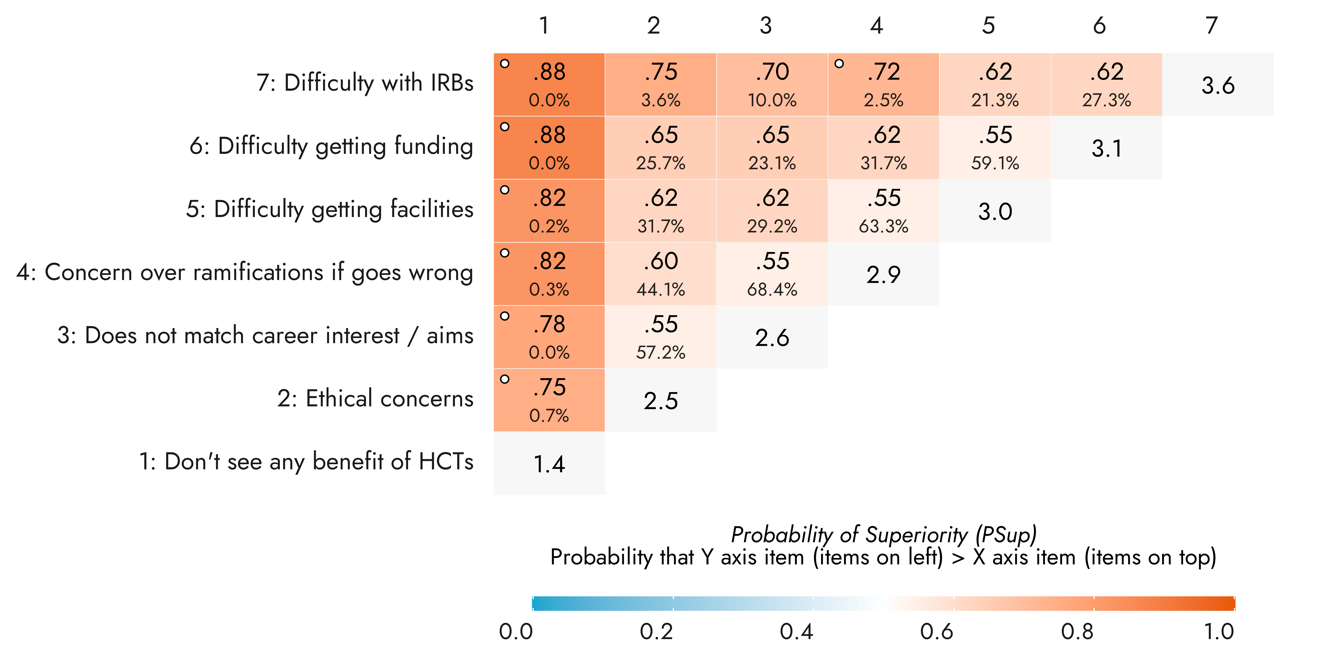
**

Figure S9. Probability of superiority for barriers to conducting an HCT.

Human Challenge Trials Study - Expert Opinion

Start of Block: Default Question Block

time_consent Timing

First Click (1)

Last Click (2)

Page Submit (3)

Click Count (4)

consent_section
**Title:** Expert Opinion - Attitudes Towards Human Challenge Trials
 **PI:** James Elsey, PhD., Jamie@rethinkpriorities.org, +1 202-681-3090.

 Downloadable version of consent form is [available here](https://rethinkpriorities.qualtrics.com/CP/File.php?F=F_a8FP6MrUOBhO3MW).

 Thank you for your interest in this study. Before taking part, you should understand the procedure. So, please read the text below. To understand this text, you must be fluent in English. If you are not fluent in English, please stop here. This survey is for people aged 18 and older. To take part, you must have been involved in conducting phase III clinical trials in infectious diseases in the past 5 years. Any research person involved in the process of running a phase III trial would be eligible (e.g., PI, statistician, research nurse, physician, recruitment manager). This survey is not for trial volunteers.

 **Project Description:** This study aims to understand the views of researchers involved in clinical trials towards a type of medical research known as a Human Challenge Trial (HCT). The concept of an HCT will be explained. Then we will ask you some questions to see what you think about them. We will also ask some basic questions about you, such as your country of residence and your role in clinical trials. The survey should take 15 minutes.

 **Expected number of participants:** About 100

 **Risks and discomforts:** There are no expected discomforts from taking part. Any study involves a potential risk for breach of confidentiality.

 **Benefits:** There is no direct benefit to you for taking part. The findings of the study may inform you about views towards HCTs in your field. You may find this interesting or useful.

 **Information about payment and cost:** There is no reward or cost to you for taking part.

 **Confidentiality:** All data collected by Rethink Priorities will be securely collected and stored. Data will be stored on a secure server protected with current firewall and antivirus software, and backed up daily. Computers are password protected and updated daily for the latest security patches. Anonymous data will be used for research purposes. It may also be placed in an open access repository for other researchers to use.

 **Voluntary participation:** Taking part is voluntary. You may choose not to fill in the survey or stop part-way through for any reason.

 **Withdrawal from the study:** To withdraw, you can choose to stop answering questions now or at any point in the survey. Simply close this web page.
  
**Questions:** If you have any questions or concerns about this study, please email James Elsey (Jamie@rethinkpriorities.org, +1 202-681-3090). If you have questions about your rights as a participant, or concerns or complaints about the research, you may contact Solutions IRB (the body that oversees our protection of study participants: +1 855-226-4472, 8:00 a.m. to 5:00 p.m. MST, Monday through Friday). You can email the IRB at participants@solutionsirb.com. This research is funded by 1DaySooner via a grant from ACX Grants.
 
**If you live outside the US:** This survey is done using Qualtrics software (US-based company). This means the information collected will be transferred outside your country to the US and stored securely on Qualtrics servers (for more information visit this [https://www.qualtrics.com/security-statement/] Qualtrics website). By volunteering to complete this survey you agree to this transfer.
 **Consent to participate in the study:** To give your consent to take part, please select the consent option below. Then continue to the next page.

consent_answer **Consent options:**

- I have read the above information and **agree to take part** in this study. (1)
- I do not consent to take part and will stop here. (2)

End of Block: Default Question Block

Start of Block: eligibility block

eligible_age Are you aged 18 years old or above?

- Yes, I am aged 18 years or above (1)
- No, I am not aged 18 years or above (2)

eligible_english Are you fluent in English?

- Yes, I am fluent in English (1)
- No, I am not fluent in English (2)

eligible_trials Have you been involved in conducting phase III clinical trials related to infectious diseases within the past 5 years?

- Yes (1)
- No (2)

End of Block: eligibility block

Start of Block: hct_desc_confirst

time_desc Timing

First Click (1)

Last Click (2)

Page Submit (3)

Click Count (4)

hct_description_cf
Please read the following text carefully: **it is very important that you understand this information in order to answer questions on the following pages.**
 
Human challenge trials are a specific type of medical research that deliberately exposes participants to infection in a controlled setting (e.g., a research hospital). This is done in order to study diseases and how the immune system responds to them. It is also done to test vaccines and treatments.

 The key difference between a human challenge trial and other types of medical research with humans is that in a challenge trial, the participant is purposely exposed to infection. In other types of medical research, participants would be given a vaccine and then return to their normal lives, where they might be exposed to infection. Studies of new treatments in other types of medical research would test out treatments on participants who happen to have become infected in their daily lives.

 Participants in human challenge trials take part voluntarily, and the possible risks of taking part are explained to them. To minimize risk, researchers select participants who they expect will not suffer severe negative effects from infection. These participants are closely monitored and given care for any symptoms. Finally, researchers also carefully choose the dose and exact type of virus or bacteria to minimize the chances of serious illness.

 Human challenge trials have been used to learn about and develop treatments and vaccines for many illnesses, including influenza, malaria, typhoid, dengue fever, and cholera. Studies in recent decades have taken place without causing serious harm to participants.
 
What are some possible drawbacks of human challenge trials?

- Human challenge trials purposely expose participants to infection, which could cause harm.
- Data from human challenge trials may be inaccurate, because the participants are not the same as the people who are most at risk from natural infection. Exactly how a person is infected in a lab might also cause a different reaction than natural infection.
- As with any type of research, there is also no guarantee that a human challenge trial will result in a successful new treatment or vaccine being found.
- It is possible that a serious negative event happening in a human challenge trial could reduce public trust in scientists and medical doctors.

What are some possible benefits of human challenge trials?

- Human challenge trials can test whether a treatment or vaccine is effective using much fewer participants than in a typical medical trial - often dozens instead of multiple hundreds or thousands of people.
- Human challenge trials can also usually be done in a much shorter time frame than other types of medical trials.
- By exposing participants to infection in a controlled setting, researchers can answer questions that may be very hard to answer in normal medical trials, such as quickly working out the best dosage or timing for a treatment.
- Speeding up how quickly vaccines and treatments can be developed may help save lives - for example, when many people are harmed or killed by a virus that has no existing treatments or vaccines.

End of Block: hct_desc_confirst

Start of Block: hct_desc_profirst

time_desc2 Timing

First Click (1)

Last Click (2)

Page Submit (3)

Click Count (4)

hct_desc_pf
Please read the following text carefully: **it is very important that you understand this information in order to answer questions on the following pages.**
 
Human challenge trials are a specific type of medical research that deliberately exposes participants to infection in a controlled setting (e.g., a research hospital). This is done in order to study diseases and how the immune system responds to them. It is also done to test vaccines and treatments.

 The key difference between a human challenge trial and other types of medical research with humans is that in a challenge trial, the participant is purposely exposed to infection. In other types of medical research, participants would be given a vaccine and then return to their normal lives, where they might be exposed to infection. Studies of new treatments in other types of medical research would test out treatments on participants who happen to have become infected in their daily lives.

 Participants in human challenge trials take part voluntarily, and the possible risks of taking part are explained to them. To minimize risk, researchers select participants who they expect will not suffer severe negative effects from infection. These participants are closely monitored and given care for any symptoms. Finally, researchers also carefully choose the dose and exact type of virus or bacteria to minimize the chances of serious illness.

 Human challenge trials have been used to learn about and develop treatments and vaccines for many illnesses, including influenza, malaria, typhoid, dengue fever, and cholera. Studies in recent decades have taken place without causing serious harm to participants.
 
What are some possible benefits of human challenge trials?

- Human challenge trials can test whether a treatment or vaccine is effective using much fewer participants than in a typical medical trial - often dozens instead of multiple hundreds or thousands of people.
- Human challenge trials can also usually be done in a much shorter time frame than other types of medical trials.
- By exposing participants to infection in a controlled setting, researchers can answer questions that may be very hard to answer in normal medical trials, such as quickly working out the best dosage or timing for a treatment.
- Speeding up how quickly vaccines and treatments can be developed may help save lives - for example, when many people are harmed or killed by a virus that has no existing treatments or vaccines.

What are some possible drawbacks of human challenge trials?

- Human challenge trials purposely expose participants to infection, which could cause harm.
- Data from human challenge trials may be inaccurate, because the participants are not the same as the people who are most at risk from natural infection. Exactly how a person is infected in a lab might also cause a different reaction than natural infection.
- As with any type of research, there is also no guarantee that a human challenge trial will result in a successful new treatment or vaccine being found.
- It is possible that a serious negative event happening in a human challenge trial could reduce public trust in scientists and medical doctors.

End of Block: hct_desc_profirst

Start of Block: comprehension_check

time_compcheck Timing

First Click (1)

Last Click (2)

Page Submit (3)

Click Count (4)

check_1 Human challenge trials:

- Purposely expose participants to infection in a controlled setting. (1)
- Study people who have been infected with an illness in their daily lives. (2)
- Challenge people on a personal level to overcome obstacles they face. (3)

check_2 Human challenge trials usually select as participants:

- People who are most vulnerable to a disease. (1)
- People who are not expected to suffer major negative effects of infection. (2)
- Any participants who are at least 16 years of age or older. (3)

check_3 The key difference between a human challenge trial and other types of medical research involving humans is:

- Other types of medical research can only study vaccines, not treatments. (1)
- Other types of medical research cannot be used to investigate mental health. (2)
- Other types of medical research involve people who are exposed to infection in their daily lives, not on purpose as part of the research. (3)

End of Block: comprehension_check

Start of Block: support_oppose

time_supopp Timing

First Click (1)

Last Click (2)

Page Submit (3)

Click Count (4)

section_1 **Section 1 of 5**

sup_vaccines Would you support or oppose using human challenge trials to help *develop new vaccines* for diseases?

- Strongly support (1)
- Support (2)
- Slightly support (3)
- Neither support nor oppose (4)
- Slightly oppose (5)
- Oppose (6)
- Strongly oppose (7)

sup_treatments Would you support or oppose using human challenge trials to help *develop new treatments* for diseases?

- Strongly support (1)
- Support (2)
- Slightly support (3)
- Neither support nor oppose (4)
- Slightly oppose (5)
- Oppose (6)
- Strongly oppose (7)

sup_knowledge Would you support or oppose using human challenge trials to help *better understand diseases*?

- Strongly support (1)
- Support (2)
- Slightly support (3)
- Neither support nor oppose (4)
- Slightly oppose (5)
- Oppose (6)
- Strongly oppose (7)

allow When appropriate precautions are taken, and trials are overseen by an independent ethical board, do you think researchers should be allowed to run human challenge trials?

- Yes (1)
- No (2)
- No, and I am against any type of medical research involving humans (3)

End of Block: support_oppose

Start of Block: importance

time_importance Timing

First Click (1)

Last Click (2)

Page Submit (3)

Click Count (4)

section_2 **Section 2 of 5**

| 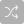 |
| --- |

importance Several things might be important in deciding whether a human challenge trial is ethically acceptable or not. Below are several things that people might consider. We want to know how important, if at all, you think each thing is in deciding whether a human challenge trial is acceptable.

|  | 1 - Not at all important (1) | 2 (2) | 3 (3) | 4 (4) | 5 (5) | 6 (6) | 7 (7) | 8 (8) | 9 (9) | 10 - Extremely important (10) |
| --- | --- | --- | --- | --- | --- | --- | --- | --- | --- | --- |
| The risk to participants who take part in the trial. (1) |  |  |  |  |  |  |  |  |  |  |
| The chance that participants would catch the illness anyway in their daily lives if not in the trial. (2) |  |  |  |  |  |  |  |  |  |  |
| How much the wider population stands to benefit from a new treatment or vaccine that might come from the trial. (3) |  |  |  |  |  |  |  |  |  |  |
| The effectiveness of any existing treatments or vaccines for the disease that is being studied in the trial. (4) |  |  |  |  |  |  |  |  |  |  |
| Whether or not effective treatments for the disease exist, and so can be given to a participant in the trial if they unexpectedly become seriously ill. (5) |  |  |  |  |  |  |  |  |  |  |
| How much the use of a human challenge trial is expected to speed up the development of a new treatment or vaccine, versus more typical procedures. (6) |  |  |  |  |  |  |  |  |  |  |
| Whether or not more typical types of experiment realistically could be used to answer the research question. (7) |  |  |  |  |  |  |  |  |  |  |
| Whether or not the main reason for participants taking part in a trial is because the pay is good, rather than wanting to help people or to advance science. (8) |  |  |  |  |  |  |  |  |  |  |
| Whether or not participants are taking part in the trial only because they really need the money. (9) |  |  |  |  |  |  |  |  |  |  |
| Whether or not participants taking part in the trial are paid according to the level of risk involved (so higher risk would mean higher payment). (10) |  |  |  |  |  |  |  |  |  |  |
| Whether or not the procedures and results of the study will be made openly available to the public. (11) |  |  |  |  |  |  |  |  |  |  |
| Whether or not participants fully understand the potential risks of taking part in the trial. (12) |  |  |  |  |  |  |  |  |  |  |
| Whether or not the trial involves a ‘placebo control group’. A placebo control means that some participants receive an inactive vaccine or treatment - for example, a sugar pill. This is often necessary to properly see if the real treatment or vaccine actually works. Participants know and agree that they may receive a placebo, but do not know whether they receive a placebo until after the study. (13) |  |  |  |  |  |  |  |  |  |  |
| Whether or not the trial is run or sponsored by a for-profit pharmaceutical company, rather than by an independent research group. (14) |  |  |  |  |  |  |  |  |  |  |
| Whether or not participants in the trial come disproportionately from disadvantaged communities or backgrounds. For example, if people in poverty, or people from a particular racial group, were much more likely to be participants than would be expected given how many such people there are in the general population. (15) |  |  |  |  |  |  |  |  |  |  |

End of Block: importance

Start of Block: direction

time_direction Timing

First Click (1)

Last Click (2)

Page Submit (3)

Click Count (4)

section_3 **Section 3 of 5**

directiondesc Please read the scenarios below. In each case, say how acceptable you think scenarios A and B are, compared to one another.

accept_availability *A human challenge trial is going to be run in one of two scenarios:*


 In *Scenario A*: There are already some treatments or vaccines for the disease.

 In *Scenario B*: There are no treatments or vaccines for the disease yet.

- A much more acceptable than B (1)
- A more acceptable than B (2)
- A slightly more acceptable than B (3)
- A and B equally acceptable (4)
- B slightly more acceptable than A (5)
- B more acceptable than A (6)
- B much more acceptable than A (7)

accept_pay4risk *A human challenge trial is going to be run in one of two scenarios:*


 In *Scenario A*: Payment for participants is not determined by the level of risk involved in taking part.

 In *Scenario B*: Payment for participants is determined by the level of risk involved in taking part (so higher risk means more payment).

- A much more acceptable than B (1)
- A more acceptable than B (2)
- A slightly more acceptable than B (3)
- A and B equally acceptable (4)
- B slightly more acceptable than A (5)
- B more acceptable than A (6)
- B much more acceptable than A (7)

accept_rescue *A human challenge trial is going to be run in one of two scenarios:*


 In *Scenario A*: Effective treatments for the disease already exist, and so can be given to a participant if they unexpectedly become seriously ill.

 In *Scenario B*: Effective treatments for the disease do not exist, and so cannot be given to a participant if they unexpectedly become seriously ill.

- A much more acceptable than B (1)
- A more acceptable than B (2)
- A slightly more acceptable than B (3)
- A and B equally acceptable (4)
- B slightly more acceptable than A (5)
- B more acceptable than A (6)
- B much more acceptable than A (7)

accept_placebo *A human challenge trial is going to be run in one of two scenarios:*


 In *Scenario A*: The trial *will not* involve a placebo control group.

 In *Scenario B*: The trial *will* involve a placebo control group.

- A much more acceptable than B (1)
- A more acceptable than B (2)
- A slightly more acceptable than B (3)
- A and B equally acceptable (4)
- B slightly more acceptable than A (5)
- B more acceptable than A (6)
- B much more acceptable than A (7)

accept_pharma *A human challenge trial is going to be run in one of two scenarios:*


 In *Scenario A*: The trial will be run by an independent research group.

 In *Scenario B*: The trial will be run by a for-profit pharmaceutical company.

- A much more acceptable than B (1)
- A more acceptable than B (2)
- A slightly more acceptable than B (3)
- A and B equally acceptable (4)
- B slightly more acceptable than A (5)
- B more acceptable than A (6)
- B much more acceptable than A (7)

End of Block: direction

Start of Block: research_block

time_research Timing

First Click (1)

Last Click (2)

Page Submit (3)

Click Count (4)

demo_description **Section 4 of 5.** This section includes some questions about your research background and interest in HCTs.

ever_conducted Have you ever conducted, or are you currently conducting, phase III clinical trials research in humans for infectious diseases?

- Yes, currently (1)
- Yes, previously (2)
- No (3)

| 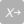 |
| --- |

country_research In which country is your research primarily based?

- United States of America (187)
- United Kingdom of Great Britain and Northern Ireland (185)
- Afghanistan (1)
- Albania (2)
- Algeria (3)
- Andorra (4)
- Angola (5)

…etc.

country_res_custom If your country did not appear in the list above or the list did not work, you can type your primary research country/countries here:

________________________________________________________________

phd Have you completed a doctorate/PhD in your research field/a related field?

- Yes (1)
- No (2)

research_context Which of the following best describes the context of your research? You may select more than one if you have joint responsibilites:

- Academic institution / Medical research hospital (1)
- Pharmaceutical industry (2)
- Other (please describe): (3) __________________________________________________

research_covers Does your clinical trials research cover any of the following areas?:

- Vaccines (1)
- Treatments (2)
- Prophylactics / preventative medicine (not including vaccines) (3)
- ⊗None of the above (4)

| 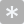 |
| --- |

research_role Which of the following fit your role responsibilities in a clinical trials research group?:

- Administrative or logistical support (1)
- Clinical trial monitor (2)
- Data manager (3)
- Ethics board member / specific ethical oversight role (4)
- Health economist (5)
- Pharmacist (6)
- Principal investigator / Chief investigator (7)
- Producer/provider of medical devices/pharmaceuticals (8)
- Qualitative researcher (9)
- Recruitment coordinator (10)
- Research nurse (11)
- Statistician (12)
- Study coordinator (13)
- Study physician (14)
- Other role (we appreciate that this list is not exhaustive of important roles in clinical trials research) (15) __________________________________________________

research_hct Have you ever conducted, or are you currently conducting, human challenge trials as part of your research on infectious diseases?

- Yes, currently (1)
- Yes, previously (2)
- No (3)

research_hct_connect Do you personally know, or have you worked with, other researchers who are conducting/have conducted human challenge trials as part of their research on infectious diseases?

- Yes (1)
- No, not that I am aware of (2)

interest_hct What level of interest do you currently have in conducting a human challenge trial as part of your research on infectious diseases?

- No interest in conducting an HCT (1)
- Low interest in conducting an HCT (2)
- Moderate interest in conducting an HCT (3)
- Considerable interest in conducting an HCT (4)
- A great deal of interest in conducting an HCT (5)

interest_hct_nobarr If there were no logistical barriers to conducting a human challenge trial as part of your research (e.g., there was funding, approval, staff, and facilities), how much interest would you have in conducting one?

- No interest in conducting an HCT (1)
- Low interest in conducting an HCT (2)
- Moderate interest in conducting an HCT (3)
- Considerable interest in conducting an HCT (4)
- A great deal of interest in conducting an HCT (5)

End of Block: research_block

Start of Block: barriers

time_barriers Timing

First Click (1)

Last Click (2)

Page Submit (3)

Click Count (4)

section_5 **Section 5 of 5**

| 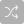 |
| --- |

hct_barriers There are many reasons why someone might not pursue a particular line of research. To what extent do the following represent a reason for you not to pursue research using human challenge trials?

|  | Not a reason for me at all (1) | A slight reason for me (2) | A moderate reason for me (3) | A strong reason for me (4) | A very strong reason for me (5) |
| --- | --- | --- | --- | --- | --- |
| Having concerns about the ethical acceptability of HCTs (1) |  |  |  |  |  |
| This type of research just doesn't match my career aims/scientific goals (2) |  |  |  |  |  |
| Difficulty getting HCTs approved by institutional review boards/ethics boards (3) |  |  |  |  |  |
| Difficulty getting funding for HCTs (4) |  |  |  |  |  |
| Difficulty getting the facilities needed to conduct HCTs (5) |  |  |  |  |  |
| Concerns over personal or professional ramifications if something went wrong in an HCT (e.g., a serious adverse event for a participant) (6) |  |  |  |  |  |
| I do not see any clear benefits of an HCT relative to more typical approaches (7) |  |  |  |  |  |

| Page Break |  |
| --- | --- |

other reasons You can use the space below if you would like to provide any other reasons why you might not pursue HCTs in your infectious disease research, or for other comments regarding this survey/topic:

________________________________________________________________

________________________________________________________________

________________________________________________________________

________________________________________________________________

________________________________________________________________

suggestions Do you have any suggestions for other researchers we might contact to assess the state of opinion in this field?:

________________________________________________________________

________________________________________________________________

________________________________________________________________

________________________________________________________________

________________________________________________________________

ending Thank you so much for taking the time to complete this survey - please be sure to click the next button to submit your responses. We really appreciate you sharing your perspectives!

End of Block: barriers

Human Challenge Trials Study

Start of Block: Default Question Block

time_consent Timing

First Click (1)

Last Click (2)

Page Submit (3)

Click Count (4)

consent_section
**Title of Project:** Attitudes Towards Human Challenge Trials
 **Primary Investigator:** James Elsey, PhD., info@rethinkpriorities.org, 202-681-3090.

 Downloadable version of consent form [available here](https://rethinkpriorities.qualtrics.com/CP/File.php?F=F_9YWzMz4UySljaHI).

 Thank you for your interest in this study. It is important that you know what taking part will involve. So, please read the text below. To understand this text, you must be fluent in English. If you are not fluent in English, then please do not continue. This survey is only for people who are 18 years or older and live in the United States.

 **Project Description:** This study aims to understand what people think about a particular type of medical research: Human Challenge Trials. You don’t need to have a background in Human Challenge Trials to fill out the survey. We will explain to you what a Human Challenge Trial is. We will then ask you some questions about what you think of them. We will also ask you some demographic information, such as your age, gender, and race. The survey should take about 10 minutes.

 **Expected number of participants:** about 2000 participants.

 **Risks and discomforts:** There are no expected discomforts from taking part in this study. There is always a potential risk for breach of confidentiality.

 **Benefits:** There is no personal benefit to you for taking part in this study.

 **Information about payment and cost:** You will be paid $1.74 for finishing the survey. You will receive the payment through the Prolific Platform after you have completed and submitted the survey. There will be no cost to you for taking part in this study.

 **Confidentiality:** In this study, we will collect your responses and your Prolific ID, but we will keep your responses separately from your Prolific ID. All data collected by Rethink Priorities in this research will be securely collected and stored. Data will be stored on a secure server protected with current firewall and antivirus software, and backed up daily. Computers are password protected and updated daily for the latest security patches. Anonymized data will be used for research. This data may also be put in an open access scientific data repository for the use of other researchers. Any identifying information (e.g., your Prolific ID) will be completely removed from any data that is shared with other researchers.

 **Voluntary participation:** Participation in this study is voluntary. You can choose not to fill in the questionnaire or stop your participation part-way through for any reason, but you will not receive compensation.

 **Withdrawal from the study:** To withdraw from the study, you can choose not to continue answering questions now. You can also stop answering questions at any point. To do that, simply close your browser. If you wish to withdraw after taking the survey, please contact James Elsey (info@rethinkpriorities.org). If you withdraw from the study within 2 weeks of submitting your questionnaire responses, then the information that you have provided will not be used for the purposes of this study.

 **Questions:** If you have any questions or concerns about this research, please contact James Elsey (info@rethinkpriorities.org). If you have questions about your rights as a research participant, or concerns or complaints about the research, you may contact the Solutions IRB (the body that oversees our protection of study participants) at 855-226-4472. Regular hoursfor the IRB are 8:00 a.m. to 5:00 p.m. MST, Monday through Friday. You can also email the IRB at participants@solutionsirb.com, or reach out via mail: Solutions IRB, PO Box 1041, Yarnell, AZ 85362.

 **Consent to participate in the study:** To provide your consent to participate in this research study, please select the consent option below, and continue to the next page.

consent_answer **Consent options:**

- I have read the above information and **agree to participate** in this study (1)
- I do not consent to participate in this study and will stop my participation at this point. (2)

| 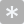 |
| --- |

prolific_id Please enter your Prolific ID below:

________________________________________________________________

End of Block: Default Question Block

Start of Block: hct_desc_confirst

time_desc Timing

First Click (1)

Last Click (2)

Page Submit (3)

Click Count (4)

hct_description_cf
Please read the following text carefully: **it is very important that you understand this information in order to answer questions on the following pages.**
 
Human challenge trials are a specific type of medical research that deliberately exposes participants to infection in a controlled setting (e.g., a research hospital). This is done in order to study diseases and how the immune system responds to them. It is also done to test vaccines and treatments.

 The key difference between a human challenge trial and other types of medical research with humans is that in a challenge trial, the participant is purposely exposed to infection. In other types of medical research, participants would be given a vaccine and then return to their normal lives, where they might be exposed to infection. Studies of new treatments in other types of medical research would test out treatments on participants who happen to have become infected in their daily lives.

 Participants in human challenge trials take part voluntarily, and the possible risks of taking part are explained to them. To minimize risk, researchers select participants who they expect will not suffer severe negative effects from infection. These participants are closely monitored and given care for any symptoms. Finally, researchers also carefully choose the dose and exact type of virus or bacteria to minimize the chances of serious illness.

 Human challenge trials have been used to learn about and develop treatments and vaccines for many illnesses, including influenza, malaria, typhoid, dengue fever, and cholera. Studies in recent decades have taken place without causing serious harm to participants.
 
What are some possible drawbacks of human challenge trials?

- Human challenge trials purposely expose participants to infection, which could cause harm.
- Data from human challenge trials may be inaccurate, because the participants are not the same as the people who are most at risk from natural infection. Exactly how a person is infected in a lab might also cause a different reaction than natural infection.
- As with any type of research, there is also no guarantee that a human challenge trial will result in a successful new treatment or vaccine being found.
- It is possible that a serious negative event happening in a human challenge trial could reduce public trust in scientists and medical doctors.

What are some possible benefits of human challenge trials?

- Human challenge trials can test whether a treatment or vaccine is effective using much fewer participants than in a typical medical trial - often dozens instead of multiple hundreds or thousands of people.
- Human challenge trials can also usually be done in a much shorter time frame than other types of medical trials.
- By exposing participants to infection in a controlled setting, researchers can answer questions that may be very hard to answer in normal medical trials, such as quickly working out the best dosage or timing for a treatment.
- Speeding up how quickly vaccines and treatments can be developed may help save lives - for example, when many people are harmed or killed by a virus that has no existing treatments or vaccines.

End of Block: hct_desc_confirst

Start of Block: hct_desc_profirst

time_desc2 Timing

First Click (1)

Last Click (2)

Page Submit (3)

Click Count (4)

hct_desc_pf
Please read the following text carefully: **it is very important that you understand this information in order to answer questions on the following pages.**
 
Human challenge trials are a specific type of medical research that deliberately exposes participants to infection in a controlled setting (e.g., a research hospital). This is done in order to study diseases and how the immune system responds to them. It is also done to test vaccines and treatments.

 The key difference between a human challenge trial and other types of medical research with humans is that in a challenge trial, the participant is purposely exposed to infection. In other types of medical research, participants would be given a vaccine and then return to their normal lives, where they might be exposed to infection. Studies of new treatments in other types of medical research would test out treatments on participants who happen to have become infected in their daily lives.

 Participants in human challenge trials take part voluntarily, and the possible risks of taking part are explained to them. To minimize risk, researchers select participants who they expect will not suffer severe negative effects from infection. These participants are closely monitored and given care for any symptoms. Finally, researchers also carefully choose the dose and exact type of virus or bacteria to minimize the chances of serious illness.

 Human challenge trials have been used to learn about and develop treatments and vaccines for many illnesses, including influenza, malaria, typhoid, dengue fever, and cholera. Studies in recent decades have taken place without causing serious harm to participants.
 
What are some possible benefits of human challenge trials?

- Human challenge trials can test whether a treatment or vaccine is effective using much fewer participants than in a typical medical trial - often dozens instead of multiple hundreds or thousands of people.
- Human challenge trials can also usually be done in a much shorter time frame than other types of medical trials.
- By exposing participants to infection in a controlled setting, researchers can answer questions that may be very hard to answer in normal medical trials, such as quickly working out the best dosage or timing for a treatment.
- Speeding up how quickly vaccines and treatments can be developed may help save lives - for example, when many people are harmed or killed by a virus that has no existing treatments or vaccines.

What are some possible drawbacks of human challenge trials?

- Human challenge trials purposely expose participants to infection, which could cause harm.
- Data from human challenge trials may be inaccurate, because the participants are not the same as the people who are most at risk from natural infection. Exactly how a person is infected in a lab might also cause a different reaction than natural infection.
- As with any type of research, there is also no guarantee that a human challenge trial will result in a successful new treatment or vaccine being found.
- It is possible that a serious negative event happening in a human challenge trial could reduce public trust in scientists and medical doctors.

End of Block: hct_desc_profirst

Start of Block: comprehension_check

time_compcheck Timing

First Click (1)

Last Click (2)

Page Submit (3)

Click Count (4)

check_1 Human challenge trials:

- Purposely expose participants to infection in a controlled setting. (1)
- Study people who have been infected with an illness in their daily lives. (2)
- Challenge people on a personal level to overcome obstacles they face. (3)

check_2 Human challenge trials usually select as participants:

- People who are most vulnerable to a disease. (1)
- People who are not expected to suffer major negative effects of infection. (2)
- Any participants who are at least 16 years of age or older. (3)

check_3 The key difference between a human challenge trial and other types of medical research involving humans is:

- Other types of medical research can only study vaccines, not treatments. (1)
- Other types of medical research cannot be used to investigate mental health. (2)
- Other types of medical research involve people who are exposed to infection in their daily lives, not on purpose as part of the research. (3)

End of Block: comprehension_check

Start of Block: support_oppose

time_supopp Timing

First Click (1)

Last Click (2)

Page Submit (3)

Click Count (4)

sup_vaccines Would you support or oppose using human challenge trials to help *develop new vaccines* for diseases?

- Strongly support (1)
- Support (2)
- Slightly support (3)
- Neither support nor oppose (4)
- Slightly oppose (5)
- Oppose (6)
- Strongly oppose (7)

sup_treatments Would you support or oppose using human challenge trials to help *develop new treatments* for diseases?

- Strongly support (1)
- Support (2)
- Slightly support (3)
- Neither support nor oppose (4)
- Slightly oppose (5)
- Oppose (6)
- Strongly oppose (7)

sup_knowledge Would you support or oppose using human challenge trials to help *better understand diseases*?

- Strongly support (1)
- Support (2)
- Slightly support (3)
- Neither support nor oppose (4)
- Slightly oppose (5)
- Oppose (6)
- Strongly oppose (7)

allow When appropriate precautions are taken, and trials are overseen by an independent ethical board, do you think researchers should be allowed to run human challenge trials?

- Yes (1)
- No (2)
- No, and I am against any type of medical research involving humans (3)

End of Block: support_oppose

Start of Block: importance

time_importance Timing

First Click (1)

Last Click (2)

Page Submit (3)

Click Count (4)

| 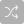 |
| --- |

importance Several things might be important in deciding whether a human challenge trial is ethically acceptable or not. Below are several things that people might consider. We want to know how important, if at all, you think each thing is in deciding whether a human challenge trial is acceptable.

|  | 1 - Not at all important (1) | 2 (2) | 3 (3) | 4 (4) | 5 (5) | 6 (6) | 7 (7) | 8 (8) | 9 (9) | 10 - Extremely important (10) |
| --- | --- | --- | --- | --- | --- | --- | --- | --- | --- | --- |
| The risk to participants who take part in the trial. (1) |  |  |  |  |  |  |  |  |  |  |
| The chance that participants would catch the illness anyway in their daily lives if not in the trial. (2) |  |  |  |  |  |  |  |  |  |  |
| How much the wider population stands to benefit from a new treatment or vaccine that might come from the trial. (3) |  |  |  |  |  |  |  |  |  |  |
| The effectiveness of any existing treatments or vaccines for the disease that is being studied in the trial. (4) |  |  |  |  |  |  |  |  |  |  |
| Whether or not effective treatments for the disease exist, and so can be given to a participant in the trial if they unexpectedly become seriously ill. (5) |  |  |  |  |  |  |  |  |  |  |
| How much the use of a human challenge trial is expected to speed up the development of a new treatment or vaccine, versus more typical procedures. (6) |  |  |  |  |  |  |  |  |  |  |
| Whether or not more typical types of experiment realistically could be used to answer the research question. (7) |  |  |  |  |  |  |  |  |  |  |
| Whether or not the main reason for participants taking part in a trial is because the pay is good, rather than wanting to help people or to advance science. (8) |  |  |  |  |  |  |  |  |  |  |
| Whether or not participants are taking part in the trial only because they really need the money. (9) |  |  |  |  |  |  |  |  |  |  |
| Whether or not participants taking part in the trial are paid according to the level of risk involved (so higher risk would mean higher payment). (10) |  |  |  |  |  |  |  |  |  |  |
| Whether or not the procedures and results of the study will be made openly available to the public. (11) |  |  |  |  |  |  |  |  |  |  |
| Whether or not participants fully understand the potential risks of taking part in the trial. (12) |  |  |  |  |  |  |  |  |  |  |
| Whether or not the trial involves a ‘placebo control group’. A placebo control means that some participants receive an inactive vaccine or treatment - for example, a sugar pill. This is often necessary to properly see if the real treatment or vaccine actually works. Participants know and agree that they may receive a placebo, but do not know whether they receive a placebo until after the study. (13) |  |  |  |  |  |  |  |  |  |  |
| Whether or not the trial is run or sponsored by a for-profit pharmaceutical company, rather than by an independent research group. (14) |  |  |  |  |  |  |  |  |  |  |
| Whether or not participants in the trial come disproportionately from disadvantaged communities or backgrounds. For example, if people in poverty, or people from a particular racial group, were much more likely to be participants than would be expected given how many such people there are in the general population. (15) |  |  |  |  |  |  |  |  |  |  |

End of Block: importance

Start of Block: direction

time_direction Timing

First Click (1)

Last Click (2)

Page Submit (3)

Click Count (4)

directiondesc Please read the scenarios below. In each case, say how acceptable you think scenarios A and B are, compared to one another.

accept_availability *A human challenge trial is going to be run in one of two scenarios:*


 In *Scenario A*: There are already some treatments or vaccines for the disease.

 In *Scenario B*: There are no treatments or vaccines for the disease yet.

- A much more acceptable than B (1)
- A more acceptable than B (2)
- A slightly more acceptable than B (3)
- A and B equally acceptable (4)
- B slightly more acceptable than A (5)
- B more acceptable than A (6)
- B much more acceptable than A (7)

accept_pay4risk *A human challenge trial is going to be run in one of two scenarios:*


 In *Scenario A*: Payment for participants is not determined by the level of risk involved in taking part.

 In *Scenario B*: Payment for participants is determined by the level of risk involved in taking part (so higher risk means more payment).

- A much more acceptable than B (1)
- A more acceptable than B (2)
- A slightly more acceptable than B (3)
- A and B equally acceptable (4)
- B slightly more acceptable than A (5)
- B more acceptable than A (6)
- B much more acceptable than A (7)

accept_rescue *A human challenge trial is going to be run in one of two scenarios:*


 In *Scenario A*: Effective treatments for the disease already exist, and so can be given to a participant if they unexpectedly become seriously ill.

 In *Scenario B*: Effective treatments for the disease do not exist, and so cannot be given to a participant if they unexpectedly become seriously ill.

- A much more acceptable than B (1)
- A more acceptable than B (2)
- A slightly more acceptable than B (3)
- A and B equally acceptable (4)
- B slightly more acceptable than A (5)
- B more acceptable than A (6)
- B much more acceptable than A (7)

accept_placebo *A human challenge trial is going to be run in one of two scenarios:*


 In *Scenario A*: The trial *will not* involve a placebo control group.

 In *Scenario B*: The trial *will* involve a placebo control group.

- A much more acceptable than B (1)
- A more acceptable than B (2)
- A slightly more acceptable than B (3)
- A and B equally acceptable (4)
- B slightly more acceptable than A (5)
- B more acceptable than A (6)
- B much more acceptable than A (7)

accept_pharma *A human challenge trial is going to be run in one of two scenarios:*


 In *Scenario A*: The trial will be run by an independent research group.

 In *Scenario B*: The trial will be run by a for-profit pharmaceutical company.

- A much more acceptable than B (1)
- A more acceptable than B (2)
- A slightly more acceptable than B (3)
- A and B equally acceptable (4)
- B slightly more acceptable than A (5)
- B more acceptable than A (6)
- B much more acceptable than A (7)

End of Block: direction

Start of Block: demo_block

time_demo Timing

First Click (1)

Last Click (2)

Page Submit (3)

Click Count (4)

demo_description We have some final general questions about you, so that we can understand who is taking our survey.

sex_og What is your sex?

- Male (1)
- Female (2)
- Other identification (3)

| 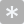 |
| --- |

age_og What is your current age in years?

________________________________________________________________

education_og What is the highest level of education you have completed?

- Some high school (1)
- Graduated from high school (Diploma/GED or equivalent) (2)
- Some college, no degree (3)
- Completed associate’s degree (4)
- Completed bachelor’s degree (5)
- Completed master’s degree (6)
- Completed professional degree beyond a bachelor’s degree (e.g., M.D., J.D.) (7)
- Completed doctorate degree (8)

income_og What is your household income? (please provide your pre-tax income)

- Under $20,000 (1)
- Between $20,000 and $49,999 (2)
- Between $50,000 and $79,999 (3)
- Between $80,000 and $99,999 (4)
- Between $100,000 and $150,000 (5)
- Over $150,000 (6)

hispanic_og Do you consider yourself to be of Hispanic, Latino, or Spanish origin?

- Yes (1)
- No, I am NOT of Hispanic, Latino, or Spanish origin (2)

race_og Which of the following best matches your racial identity?:

- American Indian or Alaska Native (1)
- Asian or Asian American (2)
- Black or African American (3)
- Native Hawaiian or Pacific Islander (4)
- White or Caucasian (5)
- Other race (6)
- Identify with two or more races (7)

state_og In which US State or District do you currently live?

▼ Alabama (1) ... Other US territory (52)

| 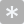 |
| --- |

zip What is your 5 digit US zipcode?

________________________________________________________________

party_raw Generally speaking, do you think of yourself as a...?

- Democrat (1)
- Republican (2)
- Independent (3)
- Other (4)
- Not sure (5)

libcon_raw Do you think of yourself as politically liberal or conservative?

- Extremely conservative (1)
- Conservative (2)
- Slightly conservative (3)
- Moderate (4)
- Slightly liberal (5)
- Liberal (6)
- Extremely liberal (7)

institution_desc We are going to name some institutions in this country (specifically, the Scientific Community, and Medicine). As far as the people running these institutions are concerned, would you say you have a great deal of confidence, only some confidence, or hardly any confidence at all in them?

conf_scicomm Scientific community

- A great deal of confidence (1)
- Only some confidence (2)
- Hardly any confidence (3)

conf_medcomm Medicine

- A great deal of confidence (1)
- Only some confidence (2)
- Hardly any confidence (3)

vaccine Have you gotten a COVID-19 vaccine, or not?

- Yes (1)
- No, but I will get it in the future (2)
- No, and I am not sure if I will get it in the future (3)
- No, and I don't plan to get it (4)

pres_approve_nonbin Do you approve or disapprove of the way Joe Biden is handling his job as President?

- **Strongly approve** (1)
- **Somewhat approve** (2)
- **Somewhat disapprove** (3)
- **Strongly disapprove** (4)
- Don't know / No opinion (5)

trust Generally speaking, would you say that most people can be trusted or that you can't be too careful in dealing with people?

- Can trust people (1)
- Can't be too careful (2)

bible Which of these statements comes closest to describing your feelings about the Bible?

- The Bible is the actual word of God and is to be taken literally, word for word (1)
- The Bible is the inspired word of God but not everything in it should be taken literally, word for word (2)
- The Bible is an ancient book of fables, legends, history, and moral precepts recorded by men (3)

spanking It is sometimes necessary to discipline a child with a good, hard spanking:

- Strongly agree (1)
- Agree (2)
- Disagree (3)
- Strongly disagree (4)

presvote_2020 For which candidate did you vote in the 2020 Presidential Election?

- Joe Biden (the Democrat) (1)
- Donald Trump (the Republican) (2)
- Other candidate (3)
- Did not vote in 2020 election, but was eligible to vote (4)
- Was not eligible to vote in 2020 election (5)

end Thank you so much - we really appreciate your answers! Be sure to click the next button/arrow below so that you are redirected back to Prolific for payment!

End of Block: demo_block
