# Supplementary material for: Ethical acceptability of human challenge trials: Consultation with the US public and with research personnel
Source: PLoS One. 2024 Oct 22;19(10):e0307808. doi: 10.1371/journal.pone.0307808 (PMC11495607; doi:10.1371/journal.pone.0307808)
Supplement: Attachment — Submitted filename: comments.docx [file pone.0307808.s002.docx]

The paper overall is well-presented. Many doubts were explained in the limitations. However, I would recommend that the authors include a socio-demographic table summarizing the characteristics of the public participants surveyed, either in the main manuscript or as extended data.
